# PO Film: An Effective Strategy for Alleviating Leaf Photo-Oxidative Damage and Boosting Photosynthesis in Potted Tree Peony Under Summer Light and Temperature Stress

**DOI:** 10.3390/plants15030448

**Published:** 2026-02-01

**Authors:** Shuangcheng Gao, Mengqiang Shi, Shuaiying Shi, Tian Shi, Xingshu Wei, Yanbing Wang, Shiqi Li, Jizhen Liu, Yuan Zhao, Guoan Shi

**Affiliations:** 1 Henan Comprehensive Utilization Engineering Technical Research Center for Peony, College of Mudan, Henan University of Science and Technology, Luoyang 471023, China; gsczml@163.com (S.G.); smqhaust@163.com (M.S.); shishuaiying789@163.com (S.S.);; 2 College of Landscape Architecture and Art, Henan Agricultural University, Zhengzhou 450002, China

**Keywords:** tree peony, facility shading control, ROS homeostasis, antioxidant defense, multiple stress conditions

## Abstract

Tree peony (*Paeonia* sect. *Moutan*) is one of the most important traditional ornamental woody flowers in China. However, its cultivation is often challenged by intense light and high temperatures during summer, leading to severe photo-oxidative damage and leaf senescence. In this study, we investigated the protective effects of polyolefin (PO) film on potted tree peony leaves under summer intense light and high temperature stress. Using tree peony ‘Luoyanghong’ as experimental material, we systematically compared the effects of two controls under natural light (CK1 and CK2, housed in separate greenhouses), single-layer PO film (PO1), and double-layer PO film (PO2) treatments. Microenvironment monitoring showed that single-layer and double-layer PO films reduced light intensity by 21% and 49%, respectively, while having limited effects on temperature. Morphological and physiological analyses indicated that PO film treatments effectively alleviated leaf yellowing and withering, maintained normal physiological morphology, and increased chlorophyll (Chl) and carotenoid (Car) content. The net photosynthetic rates of PO1 and PO2 plants were 18–36% higher than those of the control groups (CK1 and CK2). Evans blue and NBT staining revealed that PO film reduced cellular damage and reactive oxygen species (ROS) accumulation, while decreasing malondialdehyde (MDA) levels and increasing catalase (CAT) activity. Furthermore, qRT-PCR results showed that stress-responsive genes (*Hsp70*, *Hsp90*, and *ATG5*) and photosynthetic genes (*RbcS* and *RbcL*) were upregulated under PO film treatment. Principal component analysis (PCA) indicated that high light intensity, rather than temperature, was the primary factor causing leaf damage in potted tree peonies. The results show that PO film is an effective and low-cost agronomic measure, which can alleviate the intense light stress under high temperature conditions, relieve photo-oxidative damage, maintain photosynthetic performance, and increase the survival rate of potted tree peonies in summer.

## 1. Introduction

Tree peony (*Paeonia* sect. *Moutan*), a characteristic woody flower native to China [1], has been cultivated for over 2000 years. It is widely cherished around the world for its vibrant flower colors and unique fragrance. As a renowned traditional Chinese flower, the tree peony is widely cultivated industrially [2]. However, with climate change, the frequency and intensity of extreme, intense light and high temperature events are increasing in China [3], posing severe challenges to its open-field cultivation during summer. Tree peony originated in a temperate climate and prefers coolness in summer. Intense light and high temperature stress in summer often lead to a series of physiological disorders in tree peony leaves, including photoinhibition and photo-oxidative damage [4]. These are primarily manifested as chloroplast degradation, decreased net photosynthetic rate, accumulation of reactive oxygen species (ROS), and membrane lipid peroxidation damage. Ultimately, these effects cause leaf yellowing, scorched edges, and even premature defoliation [5,6]. Studies have shown that such stress can cause severe damage to the photosynthetic apparatus in tree peony leaves, and this damage is often difficult to fully repair through the plant’s own recovery mechanisms [7].

In response to intense light and high temperature stress, various physical, chemical, and biological regulation measures have been explored in agricultural practice. These approaches primarily aim at mitigating environmental severity and enhancing plant stress resistance, enabling plants to safely endure high temperature and intense light stress. Physical protective measures typically include facility-based cultivation techniques such as shading nets [8], rain-shelter facilities [9], and kaolin spray [10,11], which reduce stress intensity by modulating micro-environmental factors. Chemical regulation strategies focus on exogenous substance applications, such as spraying oxalic acid [12], 5-aminolevulinic acid (ALA) [13], salicylic acid (SA) [14], and epibrassinolide (EBR) [15]. These compounds activate the plant’s antioxidant defense system, enhance reactive oxygen species (ROS) scavenging capacity, maintain photosynthetic stability in leaves, and protect the integrity of cell membrane structures. Also, studies have shown that foliar application of 1.0 mg·L^−1^ EBR significantly increases the net photosynthetic rate of *Paeonia ostii* ‘Fengdan’, reduces malondialdehyde accumulation, and alleviates high temperature stress damage [5]. Pretreatment with 100 μmol·L^−1^ SA effectively improves heat tolerance in tree peony by enhancing osmoprotectant levels and antioxidant enzyme activities, thereby mitigating membrane lipid peroxidation [16]. Additionally, the xanthophyll cycle plays a key role in plant photoprotection, and exogenous regulatory substances may enhance light energy dissipation capacity by influencing this cycle. Although facility-based cultivation can partially alleviate environmental stress, conventional shading nets face limitations such as non-adjustable light transmittance and limited cooling effects. Meanwhile, chemical hormone regulation presents a challenge, including high costs and complex manual operations.

In recent years, polyolefin (PO) films have demonstrated potential effects in improving the micro-environment in fruit and vegetable cultivation due to their excellent light-scattering properties and controllable light transmittance [17,18,19]. The main component of the PO film is polyolefin (including polymers such as ethylene and propylene), which has the advantages of high transparency, high strength, and long service life. Experiments have indicated that PO films exhibit superior tensile and tear strength compared to conventional film materials (PE + EVA, Polyethylene + Ethylene-Vinyl Acetate Copolymers), along with a 4% higher transmittance of photosynthetically active radiation (PAR). Lettuce and cucumbers grown under PO films showed higher Chl content and yield [20]. Owing to their controllable intense light transmittance, PO films reduced the incidence of fungal diseases in tomato and pepper while maintaining yield and quality [21]. However, other studies have shown a trade-off between cooling and insulation in different PO film formulations. Although PO films offer limited cooling compared to thermal barrier films (TBF), tomatoes cultivated under PO films exhibited a higher leaf area index and biomass [22]. Despite extensive research on the regulation of plant growth by PO films, their application in enhancing stress resistance in woody ornamental plants, particularly tree peony, remains unclear.

This research utilized the tree peony ‘Luoyanghong’ as an experimental material to investigate the protective effects of PO film on potted tree peony under intense summer light and high temperature conditions. By measuring leaf morphology, photosynthetic pigment content, photosynthetic rate, degree of cellular damage, extent of membrane lipid peroxidation, and differential expression of related genes across different treatments, and employing statistical methods such as principal component analysis (PCA), the primary environmental stress factors from the interaction of intense light and high temperature conditions. This study aims to provide technical support for the successful summer management of potted tree peonies.

## 2. Results

### 2.1. Diurnal Variation Characteristics of Potted Tree Peony Growth Environment

**Figure 1:**
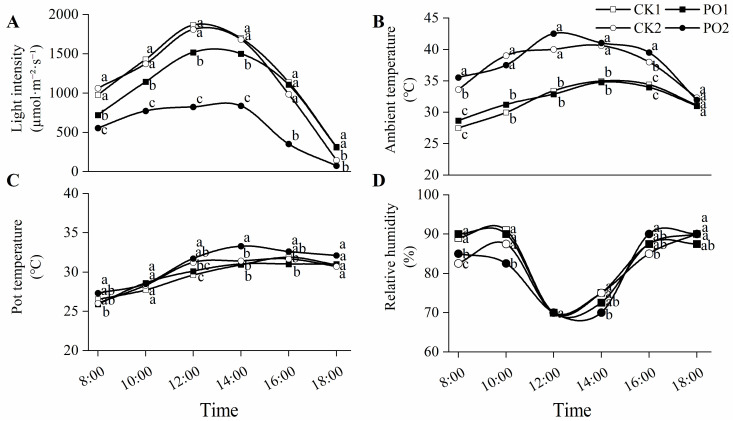
Diurnal variation in meteorological factors under light and PO film from 8:00 to 18:00 on August 6th, 2024. (**A**) The diurnal change in light intensity. (**B**) The diurnal change in ambient temperature. (**C**) The diurnal change in pot temperature. (**D**) The diurnal change in relative humidity. CK1, control of multi-span greenhouse 1; PO1, single-layer PO film treatment for multi-span greenhouse 1; CK2, multi-span greenhouse 2 control; PO2, treatment with double-layer PO film in multi-span greenhouse 2. Means with different letters indicate a significant difference (*p* < 0.05) between CK and PO film.

Early August represents the period with the highest average ten-day temperature in summer in Luoyang, China, and is a critical time for tree peonies to suffer from intense light and high temperature stress. Environmental monitoring data on 6 August (Figure 1) showed that the maximum light intensity for both CK1 and CK2 occurred at 12:00 noon, reaching 1865.22 and 1807.42 μmol⋅m^−2^⋅s^−1^, respectively, with no significant difference between them. Under single-layer PO film (PO1) and double-layer PO film (PO2) coverage, light intensity was reduced by 21% and 49%, respectively, compared to the control. On that day, the external atmospheric temperature ranged from 25.7 °C to 32.5 °C. The maximum temperatures in the two control groups also occurred at 14:00 and 12:00, reaching 35.0 °C and 40.6 °C, respectively. The maximum temperatures under PO1 and PO2 were 34.8 °C and 42.5 °C, respectively. Correspondingly, the pot temperature exceeded 30 °C after 12:00 noon. The relative humidity of the air ranged from 73% to 93%. For all treatments, the relative humidity at noon (12:00) decreased to its lowest point and then rose.

### 2.2. Changes in Leaf Morphology and Color Difference in Potted Tree Peony

**Figure 2:**
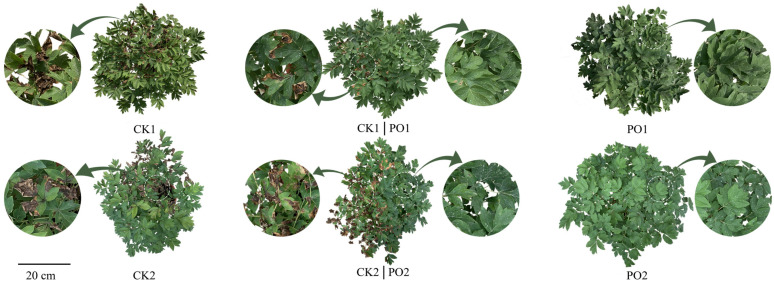
Difference in leaf morphology of potted tree peonies under natural light and PO film. CK1, Phenotype of potted tree peony leaves in the control group of multi-span greenhouse 1; PO1, Phenotype of potted tree peony leaves in the single-layer PO film treatment group in multi-span greenhouse 1. CK1|PO1, Phenotype of potted tree peony leaves at the junction of the control group and the single-layer PO film treatment in multi-span greenhouse 1. CK2, Phenotype of potted tree peony leaves in the control group in multi-span greenhouse 2. PO2: Phenotype of potted tree peony leaves in the double-layer PO film treatment group in multi-span greenhouse 2. CK2|PO2, Phenotype of potted tree peony leaves at the junction of the control group and the double-layer PO film treatment in multi-span greenhouse 2.

Distinct phenotypic differences in the leaves of potted tree peonies across treatments (Figure 2). Leaves of the control groups (CK1 and CK2) exhibited significant yellowing, with withered leaf margins and tips. In contrast, no obvious leaf scorching was observed in plants under either single-layer (PO1) or double-layer (PO2) PO film treatments. An intriguing phenomenon was noted at the junction between control and PO film-covered areas: potted plants displayed a clear “Yin-Yang face” effect. Specifically, leaves on the control side of the same plant showed yellowing, withering, and damage along the margins, while leaves on the PO film-covered side remained intact without visible injury. Therefore, both single-layer and double-layer PO film treatments effectively protected the potted tree peonies, maintaining healthy growth under intense light and high temperature stress conditions.

**Figure 3:**
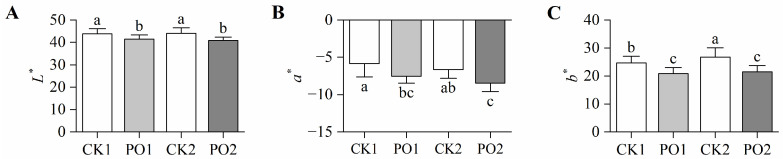
Differences in leaf color of potted tree peony under natural light and PO film. (**A**) The L* value represents the brightness of the leaf, with 0–100 indicating the range from black to white. (**B**) The a* value represents the red–green color of the leaf. A positive value indicates red, while a negative value indicates green. (**C**) The b* value represents the yellowish-blue color of the leaf. Positive values indicate yellow, while negative values indicate blue. CK1, control of multi-span greenhouse 1; PO1, single-layer PO film treatment for multi-span greenhouse 1; CK2, multi-span greenhouse 2 control; PO2, treatment with double-layer PO film in multi-span greenhouse 2. Data is presented as means ± SE of ten biological replicates. Means with different letters indicate a significant difference (*p* < 0.05) between CK and PO film.

To further investigate the effects of PO film on the leaf morphology of potted tree peonies, colorimetric analysis was conducted on leaves from different treatments. The brightness (L*) values of leaves in CK1 and CK2 were significantly higher than those in PO1 and PO2, indicating that control leaves exhibited a lighter coloration, while PO film-covered leaves showed darker hues (Figure 3A). The red–green (a*) values of leaves in PO1 and PO2 were significantly reduced by 22.31% and 21.27%, respectively, compared to CK1 and CK2 (Figure 3B). The yellow–blue (b*) value of PO1 was significantly reduced by 18.13% compared to CK1, while PO2 showed a 24.61% reduction compared to CK2 (Figure 3C). These results demonstrate that control leaves exhibited a pronounced shift toward yellow pigmentation, indicating severe chlorosis. In contrast, PO film coverage effectively maintained the green coloration of tree peony leaves.

### 2.3. Changes in Cell Activity and Damage Index of Potted Tree Peony Leaves

**Figure 4:**
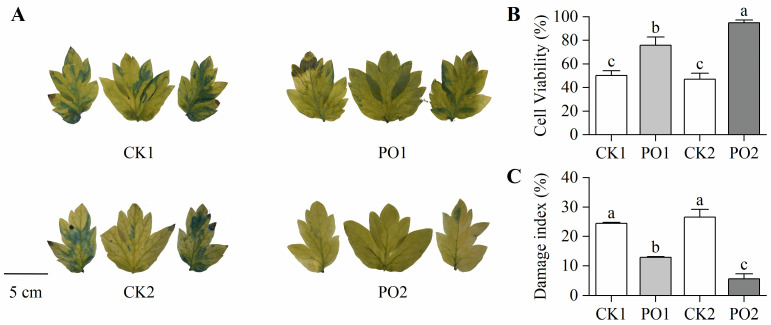
Changes in cell viability and damage index of potted tree peony leaf under natural light and PO film. (**A**) Staining of Evans blue solution in potted tree peony leaf. (**B**) Cell viability of potted tree peony leaf by Evans blue dye method. (**C**) Damage index of potted tree peony leaf. CK1, control in multi-span greenhouse 1; PO1, single-layer PO film treatment in multi-span greenhouse 1; CK2, multi-span greenhouse 2 control; PO2, treatment with double-layer PO film in multi-span greenhouse 2. Data is presented as means ± SE of ten biological replicates. Means with different letters indicate significant difference (*p* < 0.05) between CK and PO film.

In this study, Evans blue staining was performed on the leaves of potted tree peonies from different treatments. The results revealed that most leaf areas of CK1 and CK2 were stained blue, whereas leaves from PO1 and PO2 treatments showed no significant blue staining (Figure 4A), indicating that PO film coverage effectively maintained cell viability in tree peony leaves. To further quantify the degree of cellular damage, stained leaf area data were converted to determine cell viability (Figure 4B). Cell viability in PO1 and PO2 was significantly higher than in CK1 and CK2, by 51.08% and 102.41%, respectively. For a comprehensive assessment of overall plant damage across treatments, the rate of leaf scorching and withering was measured (with at least 100 pots per treatment) and converted into a damage index. The results (Figure 4C) showed that the damage index of CK1 was 1.89 times that of PO1, while CK2 exhibited a damage index 4.91 times higher than PO2. These findings demonstrate that PO film treatment significantly reduced the degree of damage at the individual leaf level in potted tree peonies.

### 2.4. Changes in Photosynthetic Pigment Content of Potted Tree Peony Leaves

**Figure 5:**
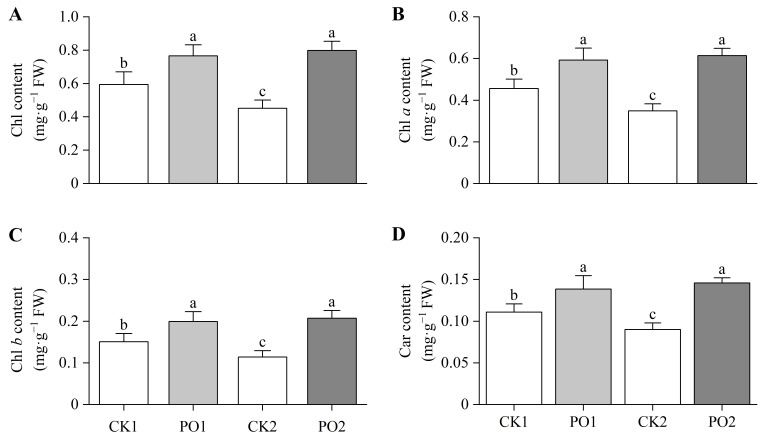
Changes in photosynthetic pigment content of potted tree peony leaves under natural light and PO film. (**A**) Total Chl content in potted tree peony leaf. (**B**) Chl *a* content in potted tree peony leaf. (**C**) Chl *b* content in potted tree peony leaf. (**D**) Carcontent in potted tree peony leaf. CK1, control in multi-span greenhouse 1; PO1, single-layer PO film treatment in multi-span greenhouse 1; CK2, multi-span greenhouse 2 control; PO2, treatment with double-layer PO film in multi-span greenhouse 2. Data is presented as means ± SE of ten biological replicates. Means with different letters indicate significant difference (*p* < 0.05) between CK and PO film.

The results of the pigment content determination of the leaves showed that the total Chlorophyll (Chl) content, Chlorophyll *a* (Chl *a*), and Chlorophyll *b* (Chl *b*) in PO1-treated potted tree peony leaves were significantly higher than those in CK1, by 28.99%, 30.12%, and 32.51%, respectively (Figure 5A–C). Similarly, PO2-treated potted tree peony leaves exhibited significantly higher total Chl, Chl *a*, and Chl *b* levels than CK2, with increases of 76.08%, 81.99%, and 77.03%, respectively. Measurement of carotenoid (Car) content revealed that PO1-treated potted tree peony leaves had 25.11% higher Car levels than CK1 (Figure 5D), while PO2-treated potted tree peony leaves showed a 61.91% increase compared to CK2.

### 2.5. Changes in Photosynthetic Characteristics of Potted Tree Peony Leaves

**Figure 6:**
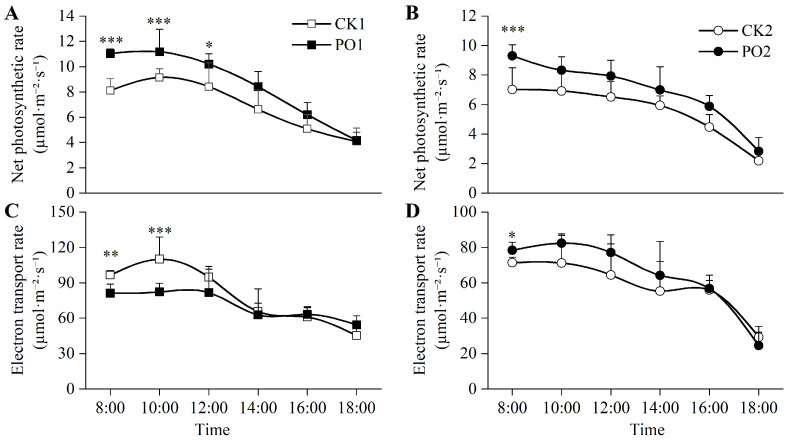
Changes in photosynthetic characteristics of potted tree peonies under natural light and PO film conditions. (**A**) Diurnal variation in potted tree peony leaf *Pn* in CK1 and PO1. (**B**) Diurnal variation in potted tree peony leaf *Pn* in CK2 and PO2. (**C**) Diurnal variation in potted tree peony leaf ETR in CK1 and PO1. (**D**) Diurnal variation in potted tree peony leaf ETR in CK2 and PO2. CK1, control in multi-span greenhouse 1; PO1, single-layer PO film treatment in multi-span greenhouse 1; CK2, multi-span greenhouse 2 control; PO2, treatment with double-layer PO film in multi-span greenhouse 2. Data is presented as means ± SE of ten biological replicates, with * *p* < 0.05, ** *p* < 0.01, *** *p* < 0.001 indicating significant differences between CK1 and PO1, and between CK2 and PO2 at the same time point.

The net photosynthetic rate (*Pn*) of leaves in PO1-treated potted tree peonies was significantly higher than that of CK1 throughout the 8:00–12:00 period, with increases of 35.88%, 22.28%, and 21.42%, respectively. Similarly, at 8:00, the *Pn* of the plants treated with PO2 was significantly higher than that of the CK2 treatment, increasing by 32.48% (Figure 6A,B).

The results of electron transport rate (ETR) showed (Figure 6C,D) that from 8:00 to 12:00, the ETR of the potted tree peony leaves in the PO1 pot was significantly higher than that of CK1, by 18.77% and 33.58%, and at 8:00, the ETR of the potted tree peony leaves in the PO2 pot was significantly higher than that of CK2 by 9.89%.

### 2.6. Changes in Oxidative Stress and Antioxidant System of Potted Tree Peony Leaf

**Figure 7:**
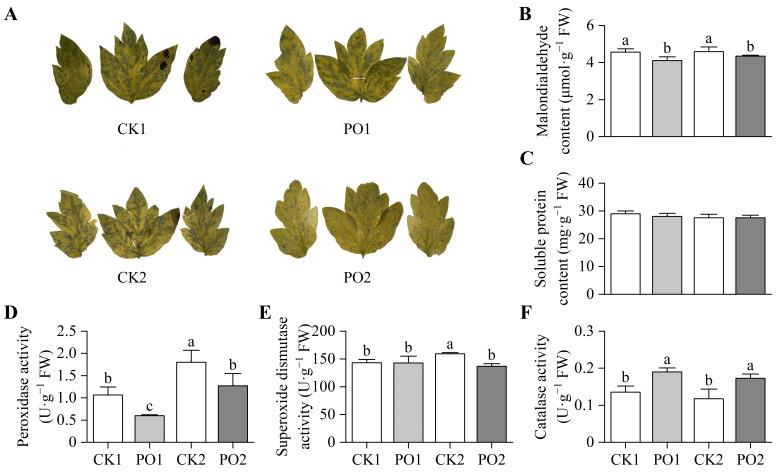
Oxidative stress and antioxidant system in leaves of potted tree peony under natural light and PO film treatments. (**A**) O_2_^·−^, Superoxide anion; (**B**) MDA, Malondialdehyde; (**C**) SP, Soluble protein; (**D**) POD, Peroxidase; (**E**) SOD, Superoxide dismutase; (**F**) CAT, Catalase. CK1, control in multi-span greenhouse 1; PO1, single-layer PO film treatment in multi-span greenhouse 1; CK2, multi-span greenhouse 2 control; PO2, treatment with double-layer PO film in multi-span greenhouse 2. Data is presented as means ± SE of ten biological replicates. Means with different letters indicate significant difference (*p* < 0.05) between CK and PO film.

There was a significant superoxide anion (O_2_^·−^) accumulation in leaves of the CK1 and CK2 treatments, whereas markedly less accumulation was observed in the PO1 and PO2 treatments (Figure 7A). Malondialdehyde (MDA) is a product accumulated after plant cell membrane damage. This study found (Figure 7B) that the MDA content in leaves of CK1 was significantly higher (by 10.91%) than that in PO1, and CK2 was significantly higher (by 5.73%) than PO2. This indicates that both PO1 and PO2 treatments significantly reduced the degree of membrane damage in tree peony leaves under environmental stress. No significant differences were observed in soluble protein (SP) content among the leaves of potted tree peonies across all treatments (Figure 7C).

The results of the activity assays of peroxidase (POD), superoxide dismutase (SOD), and catalase (CAT) enzymes indicated that the POD activity in potted tree peony leaves of CK1 was significantly higher (78.53%) than that in PO1. Similarly, the POD activity in CK2 was significantly higher (41.88%) than that in PO2 (Figure 7D). The SOD activity in CK2 was significantly higher (16.53%) than that in the other three treatments (Figure 7E). In contrast, the CAT activity in PO1 was significantly higher (40.11%) than that in CK1, and the CAT activity in PO2 was significantly higher (46.84%) than that in CK2 (Figure 7F).

### 2.7. Differential Expression of Key Enzyme Genes in Potted Tree Peony Leaves

**Figure 8:**
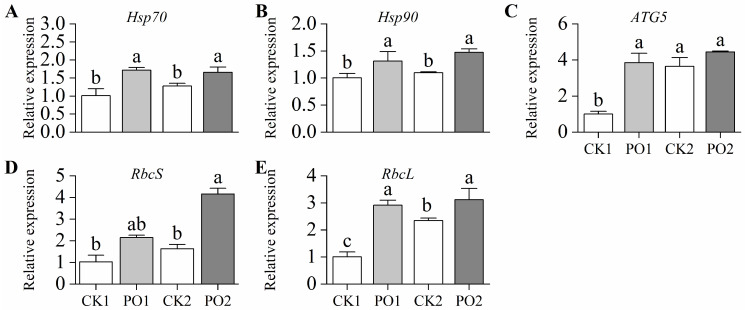
Relative expression of genes in potted tree peony under natural light and PO film. (**A**) Expression levels of heat shock protein gene *Hsp70*; (**B**) Expression levels of heat shock protein gene *Hsp90*; (**C**) Expression levels of autophagy gene *ATG5*; (**D**) Expression levels of rubisco protein synthesis gene *RbcS*; (**E**) Expression levels of rubisco protein synthesis gene *RbcL*. CK1, control in multi-span greenhouse 1; PO1, single-layer PO film treatment in multi-span greenhouse 1; CK2, multi-span greenhouse 2 control; PO2, treatment with double-layer PO film in multi-span greenhouse 2. Data is presented as means ± SE of ten biological replicates. Means with different letters indicate significant difference (*p* < 0.05) between CK and PO film.

In this study, the gene expression levels of *Hsp70* and *Hsp90* in leaves of potted tree peonies under the PO1 treatment were extremely significantly higher than those in CK1, being 1.70 and 1.30 times higher, respectively. Similarly, under the PO2 treatment, their expression levels were considerably higher than those in CK2, by 1.31 and 1.35 times, respectively (Figure 8A,B). In this study, the expression level of *ATG5* in leaves under the PO1 treatment was extremely significantly higher than that in CK1, reaching 3.83 times that of the latter. However, no significant difference was observed between PO2 and CK2 (Figure 8C).

The gene expression levels of *RbcS* and *RbcL* in the leaves of potted tree peonies under the PO1 treatment were higher than those in CK1, reaching 2.09 and 2.89 times the levels of CK1, respectively. Similarly, under the PO2 treatment, the expression levels of *RbcS* and *RbcL* were significantly higher than those in CK2, by 2.55 and 1.33 times, respectively (Figure 8D,E). These results indicate that PO film treatment significantly enhanced the synthesis of Rubisco in summer intense light and temperature conditions.

### 2.8. Correlation Analysis Between Major Physiological Indicators

**Figure 9:**
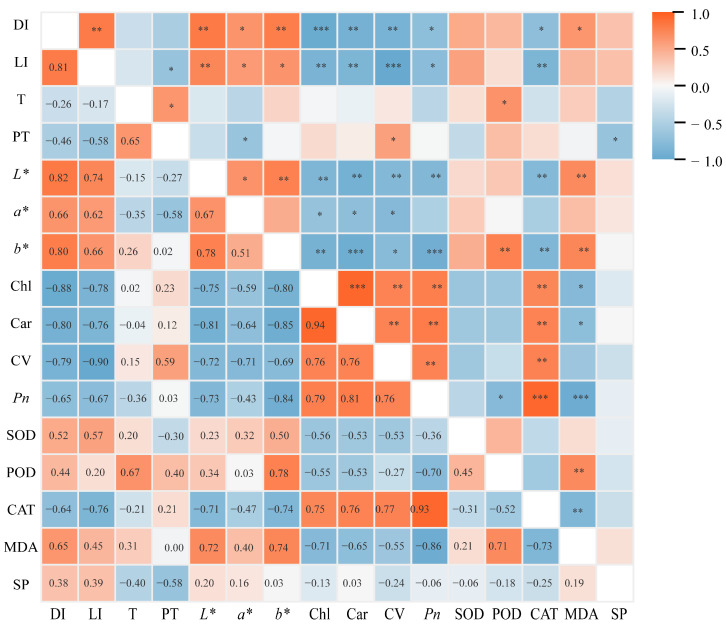
Correlation analysis between the damage index and each index. DI, Damage index. LI, Light intensity. T, Ambient temperature. PT, Pot temperature. L*, Lightness. a*, Red–green values. b*, Yellow–blue values. Chl, Chlorophyll content. Car, Carotenoid content. CV, Cell viability. *Pn*, Net photosynthetic rate. SOD, Superoxide dismutase. POD, Peroxidase. CAT, Catalase. MDA, Malondialdehyde. SP, Soluble protein. The correlations among the various indicators were analyzed using Spearman correlation.

Correlation analysis between key physiological indicators of potted tree peonies and light intensity was conducted, and the results are presented in Figure 9. The leaf damage index showed a significantly positive correlation with light intensity and colorimetric parameters (L*, a*, and b*), while exhibiting a significantly negative correlation with Chl content, car content, cell viability, Pn, and CAT activity. This indicates that the extent of leaf damage in potted tree peonies is primarily influenced by light intensity. Meanwhile, the content of Chl is significantly negatively correlated with the colorimetric values (L*, a*, and b*). The changes in leaf colorimetric values can effectively reflect the degree of leaf damage. Furthermore, the severity of damage significantly affects physiological indicators such as Chl content.

### 2.9. Comprehensive Evaluation of Leaf Damage of Potted Tree Peony

A stepwise regression analysis was performed, using the damage index as the dependent variable, to identify seven key indicators. PCA was then conducted on the damage index and these key indicators. The *KMO* (Kaiser–Meyer–Olkin) measure and Bartlett’s test results are shown in Table 1. The *KMO* value was 0.571 > 0.5, and Bartlett’s test of sphericity yielded a significance level of *p* = 0.00 (*p* < 0.01), confirming the suitability of the data for factor analysis.

**Figure 10:**
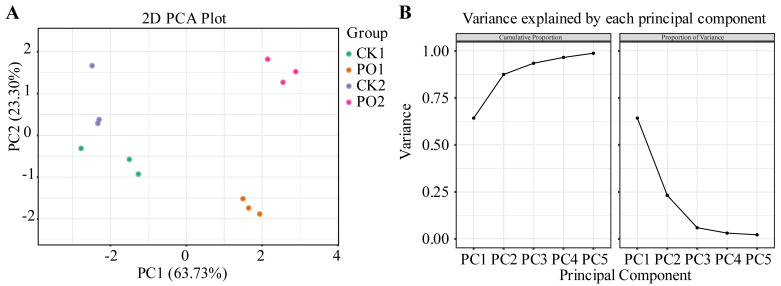
PCA of various indexes of potted tree peony. (**A**) PCA two-dimensional score plot. (**B**) The interpretive variance chart of the first 5 PC before PCA. CK1, control in multi-span greenhouse 1; PO1, single-layer PO film treatment in multi-span greenhouse 1; CK2, multi-span greenhouse 2 control; PO2, treatment with double-layer PO film in multi-span greenhouse 2.

The results of the PCA are presented in Figure 10. Two principal components (PC) were extracted, with contribution rates of 63.73% and 23.30%, respectively, and a cumulative contribution rate of 87.03%, effectively explaining the variability in the sample indicators. Further observation revealed that in the PCA graph, the first principal component could effectively separate CK1 and CK2 from PO1 and PO2, while the second principal component had a closer distance between CK1 and PO1.

Based on the PCA, a comprehensive evaluation of physiological indicators in potted tree peony leaves under different treatments was conducted (Table 2). The results showed that the PO2 treatment achieved the highest score, followed by PO1, while CK1 received the lowest growth score. This indicates that PO film treatment significantly alleviated leaf damage, maintained cellular activity, and reduced the degree of membrane lipid peroxidation.

**Figure 11:**
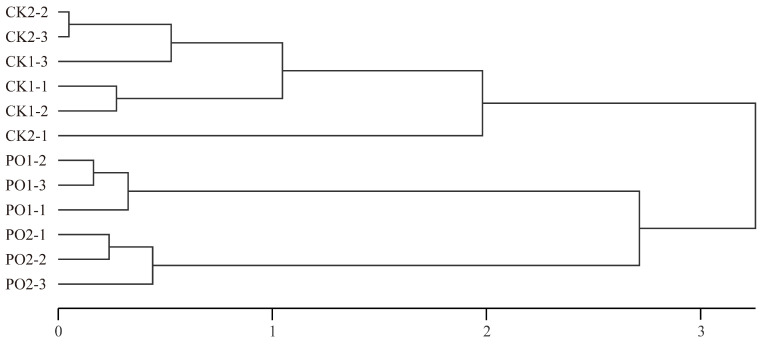
Cluster analysis of principal component scores of samples with different treatments. CK1, control in multi-span greenhouse 1; PO1, single-layer PO film treatment in multi-span greenhouse 1; CK2, multi-span greenhouse 2 control; PO2, treatment with double-layer PO film in multi-span greenhouse 2.

Hierarchical clustering was performed using the principal component (PC) scores for each sample (Figure 11). The study grouped the different experimental treatments into three distinct clusters: Cluster 1 consisted of CK1 and CK2, while Cluster 2 included PO1, and Cluster 3 included PO2. The clustering analysis clearly separated the PO film-treated groups from the control groups, demonstrating that PO film treatment effectively alleviates damage induced by high temperature and intense light stress in potted tree peony leaves. This confirms that PO film coverage is an efficient cultivation measure for mitigating high temperature and intense light stress.

## 3. Discussion

### 3.1. Light–Temperature Cross-Stress Caused Photooxidative Damage to Leaves in Summer

Our results show that under natural summer light conditions (CK1 and CK2 groups), severe photo-oxidative damage occurred in potted peony leaves, manifested specifically by leaf yellowing and wilting, reduced chlorophyll content (Figure 2 and Figure 5), and significant increases in oxidative markers such as superoxide anion (O_2_·^−^) and malondialdehyde (MDA) (Figure 7A,B). This indicates that the generally high light and high temperature stress in summer exceeds the tolerance threshold of peonies, whose optimal photosynthetic temperature range is 20–30 °C. When light radiation exceeds 1400 μmol·m^−2^·s^−1^ and the temperature rises to 40 °C, photosynthetic performance is significantly inhibited, and signs of oxidative damage appear [23], in agreement with the observed decline in *Pn* and ETR in this study (Figure 6). Under excessive illumination, the energy absorbed by the photosynthetic apparatus exceeds the amount that can be utilized for carbon fixation, causing over-reduction in the electron transport chain. This excess reductant promotes the generation of O_2_·^−^ via the Mehler reaction, subsequently triggering a chain reaction of membrane lipid peroxidation, ultimately leading to MDA accumulation and membrane injury [4,24]. This damage is corroborated by the observed decline in leaf vitality in our study and by previous research reporting the limited recovery capacity of photosystem II efficiency [25].

Furthermore, it is noteworthy that the damage caused by intense light and high temperature stress is largely irreversible. Studies have shown that the Fv/Fm ratio of leaves subjected to high temperature stress could only recover to 75.5% of its pre-stress level, and even extending the recovery time to 15 h failed to restore it completely [25]. In this study, intense light and high temperature stress induced leaf yellowing and withering in both control groups (CK1 and CK2), accompanied by reduced Chl content and diminished cell viability. These results indicate that the stress inflicted persistent damage to the photosynthetic apparatus of tree peonies during summer, ultimately impairing their normal growth.

### 3.2. Intense Light Is the First Key Environmental Factor That Causes Leaf Damage in Potted Tree Peonies

In a summer stress environment, light–temperature coupling effects often exacerbate photo-damage to potted tree peony leaves. Despite the coupled stress, the relevance and principal component analysis (PCA) of this study determined that light intensity, rather than temperature, is the main environmental factor causing leaf damage. This finding was confirmed by the PO membrane treatment: PO membranes mainly reduced light intensity (single-layer membrane reduced by 21%, double-layer membrane by 49%). Although the temperature in the connected greenhouse 2 was higher than in greenhouse 1, PO2 nonetheless significantly mitigated oxidative damage in potted peony leaves (Figure 7).

The core mechanism of intense light stress involves the accumulation of excess energy in the photosynthetic apparatus due to excessive light absorption [26,27]. In this study, the midday light intensity exceeded twice the light saturation point of tree peonies, resulting in the absorption of far more light energy by the leaves than required for carbon assimilation. When plants absorb more light energy than can be utilized for carbon assimilation, the surplus energy is dissipated through multiple pathways: (1) Heat dissipation is achieved through pathways such as non-photochemical quenching (NPQ); (2) photochemical utilization for the synthesis of ATP and NADPH; (3) reactive oxygen species (ROS) form when the above pathways are insufficient to dissipate the excess energy fully. Previous studies have demonstrated that when both the NPQ and photochemical utilization approaches approach saturation, ROS generation becomes the dominant pathway, leading to a sharp increase in intracellular ROS levels. This subsequently disrupts cellular membrane systems and chloroplast structures, causing cellular damage and reduced viability [28,29,30]. This mechanism is highly consistent with the photodamage phenomena observed in tree peony leaves under intense light conditions in the present study.

### 3.3. PO Film Is an Important Measure to Alleviate the Oxidation of Potted Tree Peony Leaves by Intense Light and High Temperature

This study confirms that covering PO membranes is an effective measure to alleviate summer stress in potted peonies. Its main benefit is reducing light intensity, thereby decreasing excess excitation energy in PSII. This reduction in energy stress directly translates to alleviation of oxidative stress. The study shows that light shading in summer can significantly delay peony aging and mitigate photosystem damage [31]. Research on other fruit trees, such as apple [32] and olive trees [33] indicates that foliar application of kaolin can effectively reduce light radiation and leaf temperature, thereby increasing net photosynthetic rate. Collectively, these results indicate that lowering light intensity can effectively reduce the leaf absorption of excess light energy, thereby lowering the excitation pressure and reactive oxygen species (ROS) production rate of photosystem II (PSII). At the same time, the antioxidant defense system is better maintained; PO-treated plants exhibit consistently higher activities of key enzymes such as SOD and CAT (Figure 7E,F), which may help scavenge the ROS produced. These findings are consistent with physiological changes observed in shaded conditions in tea plants [34].

The gene expression analysis provides deeper mechanistic insights. The upregulation of *Hsp70* and *Hsp90* under PO film treatment is a classic indicator of enhanced stress tolerance (Figure 8A,B). These molecular chaperones play crucial roles in protein folding, preventing aggregation of denatured proteins, and facilitating the refolding of proteins under stress conditions [35]. The marked upregulation of *ATG5* in PO1 plant suggests the induction of autophagy (Figure 8C), a catabolic process for recycling damaged cellular components, which is a key survival mechanism during stress [36]. Most importantly, the significant upregulation of *RbcS* and *RbcL* genes (Figure 8D,E), which encode the small and large subunits of Rubisco, underscores the improved photosynthetic capacity. Tree peonies are typical C3 plants that fix CO_2_ through the Calvin cycle. Rubisco is the key enzyme for CO_2_ fixation, and its abundance and activity are often negatively impacted by intense light and high temperature stress. The observed upregulation of *RbcS* and *RbcL* aligns with studies showing that mitigating light stress can help maintain Rubisco content and activity, thereby supporting photosynthetic performance [37]. The coordinated upregulation of these stress-responsive and photosynthetic genes strongly supports the conclusion that PO film coverage activates multiple protective pathways to enhance plant resilience.

### 3.4. Environmental Considerations and Future Perspectives for PO Film Application

While our study establishes the efficacy of PO film as a low-cost physical intervention for mitigating intense light and high temperature stress, its potential environmental impact must be considered. PO films are more durable and have higher light transmittance compared to conventional polyethylene films, potentially leading to longer service life and reduced waste [19]. In this study, no significant aging or performance degradation was observed during the use of the PO membrane. However, like other plastic films used in agriculture, their end-of-life management is crucial to avoid plastic pollution. Future adoption strategies should explore integrated waste management, including the use of recyclable PO films or the development of biodegradable alternatives, to align with sustainable agricultural practices [17].

From a research perspective, future studies should integrate multi-omics technologies (transcriptomics, proteomics, metabolomics) to systematically elucidate the complex gene regulatory networks and metabolic pathways involved in PO film-mediated stress resistance in tree peony. This will provide a more comprehensive theoretical foundation for precision facility cultivation and the development of tailored stress mitigation strategies for high-value ornamental crops.

## 4. Materials and Methods

### 4.1. Plant Materials and Treatments

**Figure 12:**
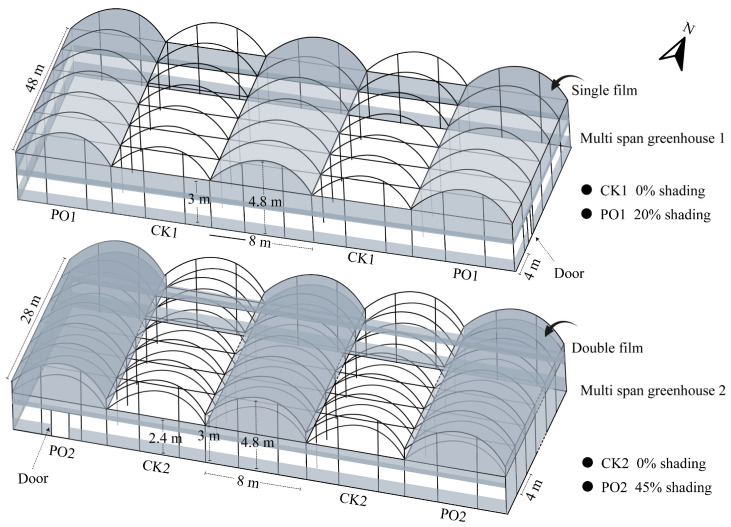
Schematic diagram of the specifications of the multi-span greenhouse used in the test.

The experiment was conducted from April to September 2024 at a potted tree peony cultivation base in Luoyang (34°43′59″ N, 112°23′23″ E, altitude 212 m), China. PO film coverage treatments were initiated after spring flowering, using PO film supplied by Shanghai Plusuck Plastic Co., Ltd. (Shanghai, China). The trial was carried out in two adjacent multi-span arched greenhouses with specifications shown in the model in Figure 12. Multi-span greenhouse 1: Alternating treatments of no roof coverage (CK1) and single-layer PO film coverage (PO1). Each north–south-oriented greenhouse contained 12 bays, with each treatment replicated three times. Side PO films were rolled up on all four sides to ensure normal ventilation. Multi-span greenhouse 2: Alternating treatments of no roof coverage (CK2) and double-layer PO film coverage (PO2). Each north–south-oriented house contained seven bays, with each treatment replicated three times. PO films on the northern and southern sides were rolled up to maintain proper ventilation. Five-year-old potted tree peony ‘Luoyanghong’ plants were moved into the greenhouses in mid-April 2024. Plants were cultivated in plastic pots with an upper diameter of 30 cm, a bottom diameter of 24 cm, and a height of 30 cm, using peat soil as the cultivation substrate, accounting for about 90% of the plastic pots. The planting density was approximately two plants per square meter, with all plants receiving automated, integrated water and fertilizer management.

A period of sustained intense light and high-temperature weather occurred from late July to early August. On August 6th, environmental condition monitoring, as well as diurnal measurements of leaf morphology and photosynthetic characteristics, were conducted. For each treatment, three different locations were randomly selected. From each location, leaves from the upper canopy of at least five potted plants were collected. The samples were transported to the laboratory on ice, washed with distilled water, blotted dry, and immediately flash-frozen in liquid nitrogen. They were then stored in a −80 °C freezer for subsequent biochemical analyses.

### 4.2. Environmental Element Records

The diurnal variation in light intensity (Photosynthetic photon flux density, PPFD) was measured using a portable photosynthetic system (Li-6800XT, LI-COR, Lincoln, NE, USA) equipped with an external light quantum sensor. The Assmann psychrometer (Model DHM3, Tianjin Zhonghua Tianyi Meteorological Instrument Co., Ltd., Tianjin, China) was employed to record air temperature and relative humidity dynamics [38]. A straight-stem geothermometer was inserted into the pot to a depth of 15 cm, positioned 5 cm from the inner pot wall, to measure the substrate temperature.

### 4.3. Leaf Morphology and Color Aberration Determination

On the sampling day, a digital camera was used to take pictures of the potted tree peonies from an overhead perspective. The images were then processed using Photoshop CC 2022. The colorimeter (SC-10, 3nh, Guangzhou, China) was used to record the color difference values (L*, a*, and b*) of the upper leaves of the potted tree peonies. For each treatment, at least 10 pots were selected for measurement.

### 4.4. Leaf Damage Index Observation

For each treatment, over 100 potted tree peonies were randomly selected. The damage level was determined by estimating the ratio of green leaf area to total leaf area for each potted tree peony plant (green leaf area/total leaf area) (Table 3), and the damage index was calculated [39,40] using the formula: Damage Index = [∑ (Number of plants per damage grade × Relative grade value)/(Total number of plants surveyed × Highest grade value)] × 100%. A higher damage index value indicates a greater degree of damage in the potted tree peonies.

### 4.5. Leaf Cell Viability Assay

Three upper leaves were randomly collected from potted tree peonies in each treatment. The samples were washed with distilled water, blotted dry, and completely immersed in 0.25% Evans blue solution for 24 h under dark conditions. After three rinses with distilled water to remove surface dye, the samples were subjected to thermal decolorization using a mixed solvent of ethanol and glycerol (anhydrous ethanol: glycerol = 9:1, *v*/*v*) at 85 °C [41]. The decolorization system was placed in a constant-temperature water bath, and the transparency of the tissues was observed every 10 min until the background color of the mesophyll cells was fully exposed (using complete whitening of the tissues as the termination criterion). The decolorized leaves were flattened in a culture dish, and digital photography was used to document the staining results.

The relative cell viability was calculated using the area-based method [42]. Image-J 1.54g software was employed to measure the non-stained leaf area and the total leaf area. The relative cell viability was determined using the following formula: Relative Cell Viability = (Non-stained Area/Total Leaf Area) × 100%.

### 4.6. Determination of Photosynthetic Pigment Content in the Leaves of Potted Tree Peonies

The content of photosynthetic pigments was determined spectrophotometrically. Briefly, leaf samples (0.1 g) from each treatment were cut into small pieces and placed in a 10 mL centrifuge tube. Pigments were extracted with 8 mL of a mixed reagent (acetone: ethanol: water = 9:9:2) and kept in darkness for 24–36 h until the leaf tissues became completely bleached. The absorbance of the extraction solution was then measured at specific wavelengths using an Ultraviolet-visible spectrophotometer (UV-4802, Unico, Shanghai, China). The contents of total chlorophyll (Chl), chlorophyll a (Chl *a*), chlorophyll *b* (Chl *b*), and carotenoids (Car) were calculated according to the standard method [43]. The pigment content was expressed as mg·g^−1^ FW.

### 4.7. Leaf Superoxide Anion (O_2_^·−^) Staining Assay

Three upper leaves were randomly collected from potted tree peonies in each treatment. The samples were washed with distilled water, blotted dry, and placed in bottles containing 0.1% NBT staining solution. The bottles were then evacuated (2 MPa) and maintained under vacuum for 20–30 min. After releasing the vacuum, the samples were incubated at room temperature for 1 h. Decolorization was performed using the thermal ethanol method, the samples were completely immersed in preheated 95% (*v*/*v*) ethanol solution at 80 °C. The decolorization solution was replaced with a fresh solution every 10 min until the green color of the tree peony leaves was substantially removed. The staining results were documented using digital photography [44].

### 4.8. Determination of Leaf Membrane Lipid Peroxidation Metabolism-Related Indexes

Leaf samples previously frozen in liquid nitrogen were used to assess membrane lipid peroxidation and antioxidant enzyme activities. The malondialdehyde (MDA) content was determined using the thiobarbituric acid (TBA) method [45]. Soluble protein (SP) content was quantified with the Coomassie Brilliant Blue G-250 staining method, using bovine serum albumin as the standard [46]. For enzyme activity assays, leaf tissue (0.2 g) was homogenized in liquid nitrogen and then extracted with 1.8 mL of ice-cold phosphate buffer (0.05 mol·L^−1^, pH 7.8) containing 1% polyvinylpyrrolidone (PVP). The homogenate was centrifuged at 10,000× *g* for 20 min at 4 °C, and the resulting supernatant was used for the following assays. Superoxide dismutase (SOD) activity was measured based on the inhibition of nitroblue tetrazolium (NBT) photoreduction [47]. Peroxidase (POD) activity was assayed using the pyrogallol method [48], and catalase (CAT) activity was determined by the ultraviolet absorption method [49]. All measurements were performed with three biological replicates per treatment. MDA content was expressed as µmol·g^−1^ FW, SP content as mg·g^−1^ FW, and enzyme activities (SOD, POD, CAT) as U·g^−1^ FW. SOD activity units (U) are defined as the amount of enzyme that inhibits NBT photoreduction by 50%. POD activity unit (U) is defined as 1 per 0.01 decrease in A470. CAT activity unit (U) is defined as 1 per 0.1 decrease in A240.

### 4.9. Determination of Leaf Photosynthetic Characteristics

On the sampling day, the measurement of photosynthetic characteristics was conducted from 08:00 to 18:00, with one measurement taken every two hours. The photosynthetic characteristics of the leaves of potted tree peonies were measured using a portable photosynthetic system (Li-6800, LI-COR, Lincoln, NE, USA). The upper functional leaves of fully expanded potted tree peonies were selected, with at least 3 plants in each treatment and each plant having more than 3 leaves. The photosynthetic effective radiation density (PPFD) was set at 1000 μmol⋅m^−2^⋅s^−1^ [50], chamber temperature is set to 30 °C, CO_2_ concentration is set to 400 μmol·mol^−1^, pressure (ΔP) is set to 0.2 kPa. Data stabilizes for 2 min before recording, and electron transport rate (ETR) data are recorded simultaneously.

### 4.10. Gene Expression by qRT-PCR

Total RNA was extracted from leaf tissues frozen in liquid nitrogen using a broad-spectrum RNA extraction kit (Beijing Nobelab Biotech Co., Ltd., Beijing, China), according to the manufacturer’s instructions. The extracted RNA is determined in concentration using a UV spectrophotometer (NanoDrop-1000, Thermo Fisher Scientific Inc., DE, USA). Using RNA as the template for reverse transcription, the obtained cDNA was used as the template to design specific primers for qRT-PCR. Subsequently, a qRT-PCR reaction was carried out using cDNA as the template. The reverse transcription kit was the HisyGo RT Red SuperMix for qPCR (+gDNA Wiper) (Nanjing Vazyme Biotech Co., Ltd., Nanjing, China), and the qRT-PCR kit was the 2 × SYBR Premix WizTaq II fluorescence quantitative PCR kit (Beijing Nobelab Biotech Co., Ltd., China). Using *ACT* (encoding actin) as the internal reference, the relative expression levels of the genes were calculated and analyzed using the 2^−ΔΔCT^ method. The specific primers for genes are listed in Table 4, including heat shock protein-related genes *Hsp70* and *Hsp90*, autophagy-related gene *ATG5*, and Rubisco protein synthesis genes *RbcS* and *RbcL*.

### 4.11. Statistical Analysis

The experimental data in this study are presented as mean ± standard error (SE). Statistical analysis was performed using SPSS 22 (IBM Corp., Somers, NY, USA). One-way analysis of variance (One-way ANOVA) followed by Duncan’s multiple range test was used for intergroup comparisons, *Pn* and ETR were compared at each time point using the *t*-test, and Spearman’s correlation analysis was applied to assess correlations. A significance threshold of *p* < 0.05 was adopted. All figures were generated using Excel, Adobe Illustrator 2024, Photoshop CC 2022, and Origin 2024.

## 5. Conclusions

This study demonstrates that single-layer and double-layer PO films reduce the light intensity inside the greenhouse by approximately 21% and 49%, respectively, thereby effectively mitigating photo-oxidative damage to the leaves of potted tree peony caused by extreme light and high temperatures during summer. Extreme light intensity is identified as the primary environmental factor leading to leaf photoinhibition. The protective physiological mechanisms conferred by PO films include the following: (1) attenuating incident light to maintain a photosynthetic steady-state in leaves; (2) enhancing antioxidant enzyme activities to preserve cellular redox homeostasis. Based on a comprehensive physiological assessment, the double-layer PO film (49% light reduction) represents a more effective cultivation strategy for enhancing the resilience of potted tree peony against summer light–heat stress. These findings provide a theoretical basis and a low-cost, practical solution for the protected cultivation of potted tree peony.

## Figures and Tables

**Table 1:** *KMO* and Bartlett’s Test.

Sufficient Karier–Meyer–Olkin (*KMO*) measures were sampled		0.571
Bartlett’s test	Approximate Chi-square	96.124
	df	28
	Sig.	0.000

**Table 2:** Comprehensive score of potted tree peony plant growth.

Treatments	F1 Score	F1 Order	F2 Score	F2 Order	Comprehensive Score	Comprehensive Order
CK1	−0.82	3	−0.17	3	−0.64	4
PO1	0.75	2	−0.53	4	0.41	2
CK2	−1.05	4	0.85	1	−0.54	3
PO2	1.12	1	−0.15	2	0.78	1

**Table 3:** Classification of leaf damage level of potted tree peony.

Green leaf area ratio (%)	0	≤20	≤40	≤60	≤80	≤95	100
Damage level	6	5	4	3	2	1	0
Damage extent	Extreme	Severe	Moderate	General	Low	Slight	No

**Table 4:** Real-time fluorescence quantitative primer information.

Gene Name	Forward Sequence (5′-3′)	Reverse Sequence (5′-3′)
*ACT*	GAGAGATTCCGTTGCCCTGA	TAGTGCAAGAGCCGTGATT
*HSP70*	GCGGTGAAGGAAATGAGAAGG	CAGGAGGAATGCCAGACAGC
*HSP90*	AACGCTCCCACCTCTGCTC	CTGAAGAACCCGACCCTCC
*ATG5*	AAGAAGGCGGAGAAGAGAA	TCAGGTGTTGTTCACTTGG
*RbcL*	AGGTGGCTGTGCTCTTAT	GCGTGAGGACATACATCTT
*RbcS*	ACCGTAAGAATGGCAGAAC	AGAAGTGAGAAGGCTTGTAG

Note: *ACT* encodes the actin gene as an internal reference gene; *Hsp70* gene encodes the Hsp70 protein; *Hsp90* gene encodes the Hsp90 protein; *ATG5* gene encodes the ATG5 protein; *RbcS* gene encodes the Rubisco small subunit; *RbcL* gene encodes the Rubisco large subunit.

## Data Availability

The original contributions presented in this study are included in the article. Further inquiries can be directed to the corresponding author.
